# Replication of Type 2 diabetes-associated variants in a Saudi Arabian population

**DOI:** 10.1152/physiolgenomics.00100.2017

**Published:** 2018-02-16

**Authors:** Ruifang Li-Gao, Salma M. Wakil, Brian F. Meyer, Nduna Dzimiri, Dennis O. Mook-Kanamori

**Affiliations:** ^1^Department of Clinical Epidemiology, Leiden University Medical Center, Leiden, the Netherlands; ^2^Department of Public Health and Primary Care, Leiden University Medical Center, Leiden, the Netherlands; ^3^Genetics Department, King Faisal Specialist Hospital and Research Center, Riyadh, Kingdom of Saudi Arabia

**Keywords:** additive model, replication, Saudi Arabia, SNPs, Type 2 diabetes

## Abstract

Over 120 Type 2 diabetes (T2D) loci have been identified from genome-wide association studies (GWAS), mainly from Caucasian populations. Very limited knowledge is available on the Saudi Arabian population. In this study, 122 previously reported T2D-related variants from 84 loci were examined in a Saudi Arabian cohort of 1,578 individuals (659 T2D cases and 919 controls). Eleven single nucleotide polymorphisms (SNPs) corresponding to nine independent loci had a *P* value <0.05. If a more stringent Bonferroni threshold of *P* = 4.1 × 10^−4^ ( = 0.05/122) were applied, none of the SNPs would have reached the significance level. Nine of the SNPs with a *P* value <0.05 showed similar odds ratios as previously described, but rs11605924 (*CRY2*) and rs9470794 (*ZFAND3*) were in the opposite direction. This study demonstrates the importance of large-scale GWAS in the Saudi Arabian population to identify ethnicity-specific disease-associated variants.

## BACKGROUND/MOTIVATION FOR THE STUDY

To date, over 120 Type 2 diabetes (T2D) loci have been identified through genome-wide association studies (GWAS) (1), mainly from Caucasian populations. The prevalence of T2D differs significantly among ethnicities because of distinct environmental and genetic factors. In the past 5 yr, more studies have focused on multiancestry GWAS for T2D. Despite a striking local diabetic epidemic in Saudi Arabia, there is limited knowledge on the genetic basis of T2D from Middle Eastern populations. Therefore, some initial evidence of overlap in T2D susceptibility loci in the Saudi Arabian population is warranted to fill in the void of genetic basis of T2D in ethnic Arabs.

## PHENOTYPE

T2D was characterized by combinations of decreased insulin secretion and sensitivity (also defined as insulin resistance). The study candidates for T2D fulfilled the World Health Organization criteria and the American Association for Diabetes Guidelines for the disease.

### 

#### Cohort details.

This study was performed in a population-based case-control study for coronary artery disease (CAD) and myocardial infarction (MI) in Saudi Arabia (2). The study population was composed of 5,668 Saudi Arabian individuals, with 2,668 CAD and MI patients cases and 3,000 controls. For the current analysis only controls were used.

#### Type of study.

Candidate SNPs.

#### Details of SNPs studied.

We examined 153 T2D-associated loci reported in Prasad and Groop (1). After imputation by the 1000G reference panel, 149 out of 153 SNPs were available. Nine SNPs were removed due to low imputation quality (imputation info <0.4), and we dropped 18 SNPs because of a global minor allele frequency (MAF) <0.1, leaving 122 variants belonging to 84 loci for analyses.

#### Analysis model.

In the current case-control analysis, additive genetic models were used to assess the associations to the risk of T2D by logistic regression, adjusted for age, sex, body mass index (BMI), and the first four principal components. The power calculation was conducted under the assumptions of 20% T2D prevalence, MAF = 0.1, genotype relative risk = 1.3, and 5% type I error rate. Around 600 cases were needed to achieve 80% power.

## RESULTS

With 659 T2D cases [mean (SD) age: 58 (12) yr, BMI: 31.0 (6.6) kg/m^2^, 53.7% men] and 919 controls [45 (16) yr, 28.3 (6.5) kg/m^2^, 54.1% men], 11 SNPs corresponding to nine independent loci had a *P* value <0.05. rs7901695, rs4506565, rs7903146 located in the *TCF7L2* gene belong to the same loci, with linkage disequilibrium (LD) of 0.97, 0.89, and 0.90, respectively, between rs7901695 and rs4506565, rs7901695 and rs7903146, and rs4506565 and rs7903146. All three SNPs in this signal had pronounced *P* values (all <0.003) and obtained similar odds ratios (ORs) as reported in the European-ancestry GWAS (ORs = 1.34, 1.32, 1.31) (see supplemental tables). (The online version of this article contains supplemental material.) In contrast, rs11605924 located in the *CRY2* gene and rs9470794 located in the *ZFAND3* gene were found to have opposite effects on T2D by estimated ORs equal to 0.80 and 0.77, respectively. If a more stringent Bonferroni threshold of *P* = 4.1 × 10^−4^ ( = 0.05/122) were applied, none of the SNPs would have reached the significance level.

## INTERPRETATION

The three variants located in the *TCF7L2* gene showed similar ORs as in the European-ancestry GWAS, suggesting the validity of the current analysis. The two SNPs with opposite effects may indicate Saudi Arabian-specific genetic information on T2D. Due to the moderate effect sizes of these T2D-associated SNPs (median OR = 1.12 in European ancestry), the current study is still underpowered, which partially explains the large amount of unreplicated SNPs (*n* = 111/122). Additionally, controls were relatively younger than the T2D cases in the current case-control study, and this age discrepancy may confound the analysis, although we adjusted for age in the multivariable model.

## GRANTS

This study was funded through Royal Cardiovascular Research Grant RAC2030012 under the King Faisal Specialist Hospital and Research Centre.

## DISCLOSURES

No conflicts of interest, financial or otherwise, are declared by the authors.

## AUTHOR CONTRIBUTIONS

R.L.-G. and D.O.M.K. analyzed data; R.L.-G. and D.O.M.K. interpreted results of experiments; R.L.-G. prepared figures; R.L.-G. drafted manuscript; R.L.-G., S.M.W., B.F.M., N.D., and D.O.M.K. edited and revised manuscript; R.L.-G., S.M.W., B.F.M., N.D., and D.O.M.K. approved final version of manuscript; S.M.W. performed experiments; B.F.M. and N.D. conceived and designed research.

## Supplemental Data

Table S1 Table S1 - .pdf (106 KB)

Table S2 Table S2 - .pdf (105 KB)
