# Supplementary material for: Replication of Type 2 diabetes-associated variants in a Saudi Arabian population
Source: Physiol Genomics. 2018 Feb 16;50(4):296–7. doi: 10.1152/physiolgenomics.00100.2017 (PMC5966803; doi:10.1152/physiolgenomics.00100.2017)
Supplement: Table S1 — pdf (106 KB) [file Table_S1.pdf]

Study genotyping / QC							SNP QC					Imputation information						Sample QC	
**Cohort Name**	**Array used and version**	**Genotype calling software**	**Related individuals (yes/no)?**	**Familial adjustment method (if applicable)**	**Population stratification assessment and adjustment**	**Analysis software version**	**MAF**	**Call Rate**	**HWE p-value**	**# SNPs analysed post-QC**	**Other filtering**	**Number of SNPs used for imputation**	**Imputation software**	**Imputation backbone, if 1000 genomes indicate the release**	**Haplotypes used for backbone**	**NCBI build**	**Chr X imputed yes/no**	**Call Rate**	**Other QC exclusions (e.g. IBS clustering, heterozygosity, other)**
Saudi CAD cohort	Affymetrix Axiom Genome-wide ASI array (Asian population)	Affymetrix Power Tools Software v.1.16	no	Exclusion based on IBS clustering (PI_HAT 0.25)	Exclusion based on PCA	SNPTEST v2	>10%	>98%	> 10-6	381635	No	381635	IMPUTE2	1000 Genomes Phase one integrated variant release v3	NA	NCBI build37	yes	>98%	exclusion based on gender mismatch; exclusion based on PCA; exclusion based on IBS clustering (PI_HAT 0.25); exclusion based on heterozygosity (>3SD heterozygosity rate)
