# Supplementary material for: Replication of Type 2 diabetes-associated variants in a Saudi Arabian population
Source: Physiol Genomics. 2018 Feb 16;50(4):296–7. doi: 10.1152/physiolgenomics.00100.2017 (PMC5966803; doi:10.1152/physiolgenomics.00100.2017)
Supplement: Table S2 — pdf (105 KB) [file Table_S2.pdf]

rsID	Locus name	Chr	Position	EA/ALT	Trait	EAF_all	EAF_cases	EAF_cons	HWE_cases	HWE_cons	OR	*P*	N_cases	N_cons	Info
rs7901695	TCF7L2(intron)	10	114754088	C/T	T2D	0.373	0.391	0.360	1.000	0.668	1.338	1.452E-03	659	919	0.851
rs4506565	TCF7L2(intron)	10	114756041	T/A	T2D	0.381	0.398	0.368	0.745	0.723	1.317	2.063E-03	659	919	0.879
rs7903146	TCF7L2(intronic/promoter)	10	114758349	T/C	T2D	0.359	0.376	0.347	0.619	0.884	1.311	2.739E-03	659	919	0.879
rs11605924	CRY2(intron)	11	45873091	A/C	T2D	0.498	0.470	0.518	0.639	0.029	0.796	4.921E-03	659	919	0.992
rs340874	PROX1(intergenic)	1	214159256	C/T	T2D	0.398	0.422	0.380	0.202	0.002	1.252	5.062E-03	659	919	0.982
rs2943641	IRS1(intergenic)	2	227093745	C/T	T2D	0.574	0.598	0.558	0.466	0.095	1.195	3.164E-02	659	919	0.948
rs13266634	SLC30A8(coding-missense)	8	118184783	C/T	T2D	0.838	0.845	0.834	0.658	0.285	1.250	3.837E-02	659	919	1.000
rs11642841	FTO(intron)	16	53845487	A/C	T2D	0.397	0.415	0.384	0.173	0.144	1.179	4.271E-02	659	919	1.000
rs560887	G6PC2/ABCB11(intron)	2	169763148	T/C	T2D	0.214	0.228	0.203	0.580	1.000	1.219	4.312E-02	659	919	1.000
rs6017317	FITM2-R3HDML-HNF4A(intergenic)	20	42946966	G/T	T2D	0.311	0.315	0.308	0.856	0.816	1.197	4.538E-02	659	919	0.944
rs9470794	ZFAND3(intron)	6	38106844	C/T	T2D	0.105	0.091	0.115	0.231	0.141	0.768	4.914E-02	659	919	0.945
rs9939609	FTO(intron)	16	53820527	A/T	T2D	0.511	0.530	0.498	0.349	0.323	1.168	5.371E-02	659	919	1.000
rs1387153	MTNR1B(intergenic)	11	92673828	T/C	T2D	0.274	0.286	0.266	0.633	0.237	1.183	6.370E-02	659	919	0.961
rs10200833	THADA(intron)	2	43673316	G/C	T2D	0.640	0.621	0.655	0.083	0.942	0.847	6.764E-02	659	919	0.854
rs11558471	SLC30A8(UTR-3)	8	118185733	A/G	T2D	0.834	0.838	0.831	1.000	0.906	1.218	6.811E-02	659	919	1.000
rs1802295	VPS26A(UTR-3)	10	70931474	A/G	T2D	0.692	0.672	0.707	0.157	0.005	0.859	7.376E-02	659	919	1.000
rs5219	KCNJ11(coding-missense)	11	17409572	T/C	T2D	0.166	0.177	0.158	0.894	0.536	1.227	7.437E-02	659	919	0.897
rs5215	KCNJ11(coding-missense)	11	17408630	C/T	T2D	0.168	0.180	0.160	1.000	0.712	1.227	7.473E-02	659	919	0.884
rs8050136	FTO(intron)	16	53816275	A/C	T2D	0.513	0.530	0.500	0.482	0.262	1.150	8.292E-02	659	919	0.998
rs4402960	IGF2BP2(intron)	3	185511687	T/G	T2D	0.357	0.369	0.348	0.242	0.942	1.154	8.760E-02	659	919	1.000
rs4812829	HNF4A(intron)	20	42989267	A/G	T2D	0.211	0.219	0.205	0.110	1.000	1.181	8.935E-02	659	919	1.000
rs8042680	PRC1(intron)	15	91521337	A/C	T2D	0.516	0.531	0.504	0.584	0.035	1.143	9.942E-02	659	919	1.000
rs10906115	CDC123/CAMK1D(intergenic)	10	12314997	A/G	T2D	0.571	0.555	0.582	0.237	0.067	0.878	1.003E-01	659	919	1.000
rs3802177	SLC30A8(UTR-3)	8	118185025	G/A	T2D	0.843	0.847	0.841	0.764	0.806	1.197	1.088E-01	659	919	0.988

rsID	Locus name	Chr	Position	EA/ ALT	Trait	EAF_ all	EAF_ cases	EAF_ cons	HWE_ cases	HWE_ cons	OR	*P*	N_cases	N_cons	Info
rs10830963	MTNR1B(intron)	11	92708710	G/C	T2D	0.237	0.247	0.229	0.916	0.709	1.167	1.283E-01	659	919	0.873
rs1801214	WFS1(coding-missense)	4	6303022	T/C	T2D	0.597	0.608	0.588	0.682	0.220	1.161	1.299E-01	659	919	0.684
rs4689388	WFS1(nearGene-5)	4	6270056	T/C	T2D	0.409	0.397	0.419	0.625	0.136	0.866	1.329E-01	659	919	0.725
rs7656416	MAEA(intron)	4	1254535	C/T	T2D	0.893	0.889	0.895	0.429	0.155	0.769	1.454E-01	659	919	0.502
rs7578326	KIAA1486/IRS1(intron of uncharacterized LOC646736)	2	227020653	A/G	T2D	0.593	0.615	0.578	0.935	0.004	1.125	1.484E-01	659	919	1.000
rs3923113	GRB14(intergenic)	2	165501849	A/C	T2D	0.629	0.635	0.625	0.801	0.121	1.130	1.525E-01	659	919	0.941
rs10010131	WFS1(intron)	4	6292915	G/A	T2D	0.596	0.606	0.588	0.625	0.220	1.152	1.539E-01	659	919	0.684
rs7403531	RASGRP1(intron)	15	38822905	T/C	T2D	0.380	0.396	0.369	0.192	0.671	1.131	1.564E-01	659	919	0.909
rs972283	KLF14(intergenic)	7	130466854	G/A	T2D	0.620	0.628	0.615	0.739	0.404	1.126	1.575E-01	659	919	0.980
rs231362	KCNQ1(intron)	11	2691471	G/A	T2D	0.560	0.550	0.567	0.694	0.638	0.837	1.616E-01	659	919	0.402
rs1359790	SPRY2(intergenic)	13	80717156	G/A	T2D	0.851	0.864	0.842	0.404	0.619	1.183	1.668E-01	659	919	0.888
rs7178572	HMG20A(intergenic)	15	77747190	A/G	T2D	0.333	0.322	0.340	0.421	1.000	0.890	1.775E-01	659	919	0.990
rs10885122	ADRA2A(intergenic)	10	113042093	G/T	T2D	0.720	0.731	0.712	0.487	0.333	1.130	1.777E-01	659	919	0.974
rs1048886	C6orf57(coding-missense)	6	71289189	G/A	T2D	0.273	0.287	0.264	0.704	0.175	1.126	1.902E-01	659	919	1.000
rs17782313	MC4R(intergenic)	18	57851097	C/T	T2D	0.269	0.282	0.260	0.178	0.607	1.126	1.950E-01	659	919	1.000
rs1470579	IGF2BP2(intron)	3	185529080	C/A	T2D	0.386	0.392	0.382	0.327	0.530	1.113	1.954E-01	659	919	1.000
rs6446482	WFS1(intron)	4	6295693	G/C	T2D	0.593	0.602	0.587	0.684	0.220	1.136	1.981E-01	659	919	0.678
rs702634	ARL15(intron)	5	53271420	A/G	T2D	0.804	0.811	0.799	0.702	0.148	1.144	1.987E-01	659	919	0.917
rs7607980	COBLL1(coding-missense)	2	165551201	T/C	T2D	0.798	0.806	0.793	0.532	0.158	1.139	1.994E-01	659	919	0.988
rs6467136	GCC1-PAX4(intergenic)	7	127164958	G/A	T2D	0.488	0.480	0.493	0.391	0.055	0.904	2.027E-01	659	919	0.991
rs4430796	HNF1B(intron)	17	36098040	G/A	T2D	0.628	0.637	0.622	0.238	0.726	1.136	2.072E-01	659	919	0.659
rs1531343	HMGA2(intron of pseudogene)	12	66174894	C/G	T2D	0.250	0.260	0.243	0.311	0.471	1.123	2.240E-01	659	919	0.972
rs10965250	CDKN2A/2B(intergenic)	9	22133284	G/A	T2D	0.779	0.788	0.772	0.294	0.851	1.124	2.295E-01	659	919	1.000
rs17168486	DGKB(intergenic)	7	14898282	T/C	T2D	0.131	0.137	0.127	0.509	0.460	1.160	2.317E-01	659	919	0.921
rs3786897	PEPD(intron)	19	33893008	A/G	T2D	0.492	0.503	0.484	0.435	0.291	1.140	2.395E-01	659	919	0.509

rsID	Locus name	Chr	Position	EA/ ALT	Trait	EAF_ all	EAF_ cases	EAF_ cons	HWE_ cases	HWE_ cons	OR	*P*	N_cases	N_cons	Info
rs13389219	GRB14(intergenic)	2	165528876	C/T	T2D	0.600	0.603	0.598	0.290	0.074	1.103	2.415E-01	659	919	0.940
rs896854	TP53INP1(intron)	8	95960511	T/C	T2D	0.517	0.508	0.524	0.160	0.644	0.908	2.444E-01	659	919	0.971
rs553668	ADRA2A(UTR-3)	10	112839579	A/G	T2D	0.200	0.208	0.194	0.344	0.340	1.120	2.654E-01	659	919	0.930
rs243021	BCL11A(intergenic)	2	60584819	A/G	T2D	0.549	0.537	0.557	0.754	0.109	0.914	2.819E-01	659	919	0.894
rs2796441	TLE1(intergenic)	9	84308948	G/A	T2D	0.652	0.673	0.637	0.657	0.475	1.099	2.947E-01	659	919	0.889
rs7177055	HMG20A(intergenic)	15	77832762	A/G	T2D	0.636	0.644	0.630	0.497	0.832	1.101	3.053E-01	659	919	0.804
rs2383208	CDKN2A/2B(intergenic)	9	22132076	A/G	T2D	0.757	0.765	0.752	0.745	0.537	1.101	3.060E-01	659	919	1.000
rs12970134	MC4R(intergenic)	18	57884750	A/G	T2D	0.249	0.257	0.243	0.308	0.720	1.101	3.077E-01	659	919	1.000
rs7305618	HNF1A(intergenic)	12	121402932	C/T	T2D	0.785	0.777	0.790	0.573	0.134	0.903	3.124E-01	659	919	0.923
rs10811661	CDKN2B(intergenic)	9	22134094	T/C	T2D	0.781	0.788	0.776	0.351	0.569	1.100	3.247E-01	659	919	1.000
rs8108269	GIPR(intergenic)	19	46158513	G/T	T2D	0.390	0.392	0.388	0.744	0.728	1.115	3.667E-01	659	919	0.461
rs35767	IGF1(nearGene-5)	12	102875569	G/A	T2D	0.845	0.852	0.839	0.164	0.140	1.105	3.672E-01	659	919	1.000
rs4275659	MPHOSPH9(intron)	12	123447928	C/T	T2D	0.542	0.556	0.532	0.875	0.029	1.081	3.832E-01	659	919	0.813
rs7756992	CDKAL1(intron)	6	20679709	G/A	T2D	0.290	0.292	0.288	0.257	0.001	1.075	4.104E-01	659	919	0.979
rs10501320	MADD(intron)	11	47293799	G/C	T2D	0.796	0.795	0.796	0.472	0.839	1.091	4.235E-01	659	919	0.853
rs515071	ANK1(intron)	8	41519462	G/A	T2D	0.730	0.723	0.735	0.625	0.309	0.929	4.264E-01	659	919	0.947
rs7501939	HNF1B(intron)	17	36101156	T/C	T2D	0.517	0.519	0.516	0.815	0.741	1.081	4.336E-01	659	919	0.643
rs2206734	CDKAL1(intron)	6	20694884	T/C	T2D	0.236	0.241	0.232	0.395	0.166	1.075	4.411E-01	659	919	1.000
rs12779790	CDC123,CAMK1D(intergenic)	10	12328010	G/A	T2D	0.143	0.143	0.143	0.148	0.497	0.903	4.510E-01	659	919	0.686
rs9505118	SSR1-RREB1(intron)	6	7290437	A/G	T2D	0.610	0.610	0.610	0.566	0.945	1.063	4.586E-01	659	919	0.999
rs2191349	DGKB/TMEM195(intergenic)	7	15064309	T/G	T2D	0.493	0.492	0.494	0.160	0.947	0.942	4.623E-01	659	919	0.982
rs4523957	SRR(nearGene-5)	17	2208899	T/	T2D	0.526	0.519	0.532	0.938	0.947	0.938	4.645E-01	659	919	0.861
rs7138803	BCDIN3D/FAIM2(intergenic)	12	50247468	A/G	T2D	0.329	0.324	0.333	0.212	0.074	0.936	4.791E-01	659	919	0.801

rsID	Locus name	Chr	Position	EA/ ALT	Trait	EAF_ all	EAF_ cases	EAF_ cons	HWE_ cases	HWE_ cons	OR	*P*	N_cases	N_cons	Info
rs7766070	CDKAL1(intron)	6	20686573	A/C	T2D	0.241	0.244	0.239	0.292	0.084	1.068	4.798E-01	659	919	0.983
rs6815464	MAEA(intron)	4	1309901	C/G	T2D	0.894	0.893	0.895	0.533	0.474	0.881	4.870E-01	659	919	0.500
rs7647305	SFRS10(intergenic)	3	185834290	C/T	T2D	0.765	0.763	0.767	0.067	0.711	0.930	4.883E-01	659	919	0.810
rs5015480	HHEX(intergenic)	10	94465559	C/T	T2D	0.293	0.297	0.291	0.025	0.424	1.061	4.940E-01	659	919	1.000
rs8090011	LAMA1(intron)	18	7068462	G/C	T2D	0.360	0.363	0.358	1.000	0.667	1.071	4.977E-01	659	919	0.691
rs1797912	PPARG(intron)	3	12470239	A/C	T2D	0.648	0.653	0.644	0.864	0.472	1.066	5.012E-01	659	919	0.786
rs516946	ANK1(intron)	8	41519248	C/T	T2D	0.744	0.737	0.749	0.841	0.379	0.939	5.022E-01	659	919	0.937
rs459193	ANKRD55(intergenic)	5	55806751	G/A	T2D	0.483	0.475	0.489	0.815	0.075	0.951	5.390E-01	659	919	0.980
rs11257655	CDC123/CAMK1D(intergenic)	10	12307894	C/T	T2D	0.825	0.826	0.824	0.413	0.088	1.070	5.481E-01	659	919	0.849
rs4712523	CDKAL1(intron)	6	20657564	G/A	T2D	0.295	0.299	0.292	0.710	0.002	1.053	5.555E-01	659	919	1.000
rs4607103	ADAMTS9-AS2(intron)	3	64711904	C/T	T2D	0.495	0.491	0.498	0.043	0.742	0.958	5.901E-01	659	919	1.000
rs7593730	RBMS1/ITGB6(intronic)	2	161171454	C/T	T2D	0.819	0.824	0.816	0.178	0.660	1.059	5.960E-01	659	919	0.904
rs7754840	CDKAL1(intron)	6	20661250	C/G	T2D	0.291	0.295	0.288	0.572	0.010	1.047	6.000E-01	659	919	0.998
rs391300	SRR(intron)	17	2216258	G/A	T2D	0.494	0.501	0.489	0.755	0.029	1.047	6.043E-01	659	919	0.788
rs10946398	CDKAL1(intron)	6	20661034	C/A	T2D	0.291	0.295	0.288	0.512	0.008	1.046	6.057E-01	659	919	1.000
rs11071657	FAM148B(intergenic)	3	62433962	A/G	T2D	0.683	0.693	0.676	0.100	0.452	1.046	6.103E-01	659	919	0.916
rs11634397	ZFAND6(intergenic)	15	80432222	G/A	T2D	0.556	0.548	0.562	0.034	0.422	0.961	6.266E-01	659	919	0.928
rs16861329	ST6GAL1(intron)	3	186666461	G/A	T2D	0.869	0.868	0.871	0.497	0.381	0.942	6.283E-01	659	919	0.967
rs10814916	GLIS3(intron)	9	4293150	C/A	T2D	0.629	0.637	0.624	0.064	0.006	1.040	6.396E-01	659	919	0.918
rs10923931	NOTCH2(intron)	1	120517959	T/G	T2D	0.116	0.120	0.113	0.853	0.005	1.059	6.456E-01	659	919	1.000
rs11708067	ADCY5(intron)	3	123065778	A/G	T2D	0.846	0.852	0.841	0.087	0.062	1.043	7.120E-01	659	919	0.887
rs1535500	KCNK16(coding-missense)	6	39284050	T/G	T2D	0.645	0.631	0.655	0.933	0.068	0.966	7.135E-01	659	919	0.789
rs10886471	GRK5(intron)	10	121149403	C/T	T2D	0.554	0.553	0.555	0.875	0.789	0.964	7.138E-01	659	919	0.680
rs6780569	UBE2E2(intergenic)	3	23198484	G/A	T2D	0.865	0.873	0.860	0.859	0.013	0.957	7.200E-01	659	919	0.949
rs7961581	TSPAN8, LGR5(LGR5)	12	71663102	C/T	T2D	0.304	0.291	0.314	0.706	0.400	0.970	7.312E-01	659	919	1.000
rs2296172	MACF1(missense)	1	39835817	G/A	T2D	0.132	0.129	0.135	0.297	0.482	0.962	7.443E-01	659	919	1.000

rsID	Locus name	Chr	Position	EA/ ALT	Trait	EAF_ all	EAF_ cases	EAF_ cons	HWE_ cases	HWE_ cons	OR	*P*	N_cases	N_cons	Info
rs174550	FADS1(intron)	11	61571478	T/C	T2D	0.738	0.742	0.734	0.308	0.002	1.031	7.444E-01	659	919	0.996
rs7018475	CDKN2A/2B(intergenic)	9	22137685	G/T	T2D	0.376	0.388	0.368	0.413	0.202	1.026	7.558E-01	659	919	1.000
rs2028299	AP3S2(UTR-3)	15	90374257	C/A	T2D	0.213	0.211	0.214	0.483	0.077	1.030	7.644E-01	659	919	0.977
rs6795735	ADAMTS9-AS2(intron)	3	64705365	C/T	T2D	0.762	0.761	0.762	0.830	0.466	0.973	7.722E-01	659	919	0.989
rs1111875	HHEX/IDE(intergenic)	10	94462882	C/T	T2D	0.719	0.719	0.719	0.148	0.462	0.976	7.818E-01	659	919	1.000
rs7034200	GLIS3(intron)	9	4289050	A/C	T2D	0.613	0.619	0.609	0.038	0.004	1.021	7.939E-01	659	919	0.962
rs6808574	LPP(intergenic)	3	187740523	C/T	T2D	0.668	0.667	0.669	0.162	0.655	1.021	8.040E-01	659	919	1.000
rs4502156	VPS13C/C2CD4A/B(intergenic)	15	62383155	T/C	T2D	0.553	0.557	0.551	0.114	0.006	0.980	8.041E-01	659	919	0.898
rs780094	GCKR(intron)	2	27741237	C/T	T2D	0.629	0.618	0.637	0.364	1.000	1.020	8.158E-01	659	919	0.981
rs849134	JAZF1(intron)	7	28196222	A/G	T2D	0.616	0.602	0.627	0.004	0.138	0.984	8.396E-01	659	919	0.998
rs243088	BCL11A(intergenic)	2	60568745	T/A	T2D	0.630	0.632	0.629	0.093	0.672	0.983	8.433E-01	659	919	0.919
rs864745	JAZF1(intron)	7	28180556	T/C	T2D	0.616	0.602	0.627	0.005	0.159	0.985	8.471E-01	659	919	1.000
rs10757282	CDKN2A/2B(intergenic)	9	22133984	C/T	T2D	0.461	0.465	0.459	0.060	0.232	0.987	8.658E-01	659	919	0.989
rs11063069	CCND2(intergenic)	12	4374373	G/A	T2D	0.266	0.268	0.264	0.028	0.269	0.983	8.790E-01	659	919	0.663
rs6813195	TMEM154(intergenic)	4	153520475	C/T	T2D	0.595	0.597	0.593	0.571	0.151	0.989	9.031E-01	659	919	0.825
rs17584499	PTPRD(intron)	9	8879118	T/C	T2D	0.207	0.204	0.209	0.471	0.162	0.987	9.100E-01	659	919	0.774
rs2877716	ADCY5(intron)	3	123094451	C/T	T2D	0.827	0.829	0.825	0.096	0.085	0.989	9.156E-01	659	919	0.938
rs7957197	OASL/TCF1/HNF1A(intron of OASL)	12	121460686	T/A	T2D	0.855	0.858	0.853	0.074	0.358	1.010	9.316E-01	659	919	0.859
rs1153188	DCD(intergenic)	12	55098996	A/T	T2D	0.753	0.748	0.758	0.407	0.322	0.992	9.358E-01	659	919	0.989
rs831571	PSMD6(intergenic)	3	64048297	C/T	T2D	0.856	0.856	0.857	0.635	0.279	0.993	9.548E-01	659	919	0.966
rs564398	CDKN2A/2B(intergenic)	9	22029547	T/C	T2D	0.822	0.819	0.823	0.599	0.573	1.005	9.605E-01	659	919	1.000
rs4457053	ZBED3(intron of ZBED3-AS1)	5	76424949	G/A	T2D	0.348	0.352	0.346	0.200	0.273	0.996	9.635E-01	659	919	0.875
rs7041847	GLIS3(intron)	9	4287466	A/G	T2D	0.663	0.664	0.663	0.081	0.002	1.003	9.668E-01	659	919	0.980
rs7172432	C2CD4A/B(intergenic)	15	62396389	A/G	T2D	0.556	0.561	0.553	0.057	0.019	1.002	9.841E-01	659	919	0.950

rsID	Locus name	Chr	Position	EA/ ALT	Trait	EAF_ all	EAF_ cases	EAF_ cons	HWE_ cases	HWE_ cons	OR	*P*	N_cases	N_cons	Info

Abbreviations: rsID - dbSNP rsID; Locus name - gene (location of the variants); Chr - chromosome; Position - Build 37 position; EA/ALT - Effect allele and alternative allele; Trait - outcome; EAF_all - Effect allele frequency of all cases and controls; EAF_cases - Effect allele frequency of all cases; EAF_cons - Effect allele frequency of all controls; HWE_cases - hardy-Weinberg equilibrium p-values among cases; HWE_cons - hardy-Weinberg equilibrium p-values among controls; OR - odds ratio; P - P-value for association; N_case - number of cases analysed; N_cons - number of controls analysed; Info - imputation quality. The SNPs with P-values <0.05 were highlighted with yellow.
